# Prognostic significance of survivin in rectal cancer patients treated with surgery and postoperative concurrent chemo-radiation therapy

**DOI:** 10.18632/oncotarget.10445

**Published:** 2016-07-06

**Authors:** Jeong Il Yu, Hyebin Lee, Hee Chul Park, Doo Ho Choi, Yoon-La Choi, In-Gu Do, Hee Cheol Kim, Woo Yong Lee, Seong Hyeon Yun, Yong Beom Cho, Jung Wook Huh, Yoon Ah Park, Young Suk Park, Joon Oh Park, Seung Tae Kim, Won Park

**Affiliations:** ^1^ Department of Radiation Oncology, Samsung Medical Center, Sungkyunkwan University School of Medicine, Seoul, Korea; ^2^ Department of Pathology, Samsung Medical Center, Sungkyunkwan University School of Medicine, Seoul, Korea; ^3^ Department of Surgery, Samsung Medical Center, Sungkyunkwan University School of Medicine, Seoul, Korea; ^4^ Department of Medicine, Samsung Medical Center, Sungkyunkwan University School of Medicine, Seoul, Korea; ^5^ Department of Radiation Oncology, Kangbuk Samsung Hospital, Sungkyunkwan University School of Medicine, Seoul, Korea; ^6^ Department of Pathology, Kangbuk Samsung Hospital, Sungkyunkwan University School of Medicine, Seoul, Korea

**Keywords:** rectal neoplasm, total mesorectal excision, radiotherapy, survivin, prognosis

## Abstract

**Background & Aims:**

This study is designed to investigate the expression of survivin and p53 in human rectal cancer tissues and analyze associations between expression and clinical outcomes in terms of disease recurrence and survival duration.

**Results:**

During follow-up (median 119.0, range 6.6 to 161.3 months), tumor recurrence was detected in 50 patients (43.1%), and local recurrence developed as a first failure site in 13 patients (11.2%). Positive immunostaining of nuclear and cytoplasmic survivin was observed in about one quarter of patients, and about half of all patients had positive staining for p53. Both survivin and p53 were significant prognostic factors of disease-free survival in the univariate analyses, but only survivin remained a significant prognostic factor in the multivariate analysis.

**Methods:**

We performed a retrospective study with 116 locally advanced rectal cancer patients who underwent total mesorectal excision (TME) followed by postoperative concurrent chemo-radiation therapy (CCRT). Immunohistochemical staining was conducted using antibodies for survivin or p53, and their expression was analyzed using an individual score that combined the percentage of positive cells and staining intensity.

**Conclusions:**

Overexpression of nuclear and cytoplasmic survivin in locally advanced rectal cancer patients was associated with a higher recurrence rate in rectal cancer patients treated with TME followed by postoperative CCRT.

## INTRODUCTION

There has been significant evolution in therapeutic strategies for locally advanced rectal cancer over the last few decades with dramatic improvements in outcome. Several randomized trials in rectal cancer patients have proven the benefit of multimodality treatment consisting of total mesorectal excision (TME) and concurrent chemo-radiation therapy (CCRT) [2–6]. According to the results of recent prospective trials, patients with locally advanced rectal cancer experienced 3-year disease-free survival (DFS) rates of 62.9% to 72.5% after multimodality treatments [7, 8].

Although favorable outcomes have been reported consistently in those patients, there is a spectrum of treatment outcomes. Identifying patients with good prognosis and a likely high risk of recurrence in order to adapt patient-specific customized treatments remains challenging. This risk adaptive strategy could be especially crucial in patients who have a low risk of recurrence, and there is controversy about the necessity of adjuvant treatment, such as in patients with upper rectum confined tumors and/or a small amount of perirectal lymph node involved tumors. There is further controversy in patients confirmed with pathologic T3N0 after TME. More accurate prognostic factors of recurrence and survival are greatly needed for locally advanced rectal cancer.

Several factors have been suggested as possible prognostic factors of recurrence and/or survival, including clinical factors [9, 10], radiologic findings [11], and molecular markers [12, 13]. The discovery of specific biomarkers, such as onco-proteins and/or genes that could be used as selective targets of a drug, may help clinicians conduct more individually tailored treatments by risk-adapted and/or novel therapeutic approaches.

Apoptosis is the multistep complex process leading to programmed cell death. It is widely accepted that disturbances in apoptosis are very important in cancer development, progression, treatment resistance, and recurrence [14]. There are many apoptotic regulators that have been identified as prognostic factors. Among these molecular markers, survivin and p53 have been actively investigated in many cancer types, including breast cancer, non-small cell lung cancer, and colorectal cancer [15–17]. They are also known to play a critical role in apoptotic pathways and cell cycle control in mammalian cells [18]. An inverse relationship between survivin and normal p53 has been suggested, where the expression of survivin and mutation in p53 might relate to tumor biology through oncogenesis and tumor growth [16].

In the present study, we investigated the expression of survivin and p53 using immunohistochemistry (IHC) in human rectal cancer tissues obtained from surgical specimens, and analyzed associations between expression and clinical outcomes in terms of disease recurrence and survival duration.

## RESULTS

### Patient and tumor characteristics

The presentby the Samsung Medical Center Institutional Review Board (IRB No. 2016-05-116). Patient and treatment characteristics at baseline are given in Table 1. The median age was 55 years (range, 30 to 75 years), and more than half of the patients were male (68 patients, 58.6%). The median CEA level at initial diagnosis was 2.9 ng/ml (range, 1 to 43 ng/ml). Most of the patients underwent low anterior resection (LAR, 95 patients, 81.9%), and others received abdomino-perineal resection (APR, 21 patients, 18.1%). All patients enrolled in this study had stage III disease based on pathologic results from surgical specimens.

**Table 1:** Patient and tumor characteristics

Characteristics		No. of patients (%)
Age (years)	Median (range)	55 (30 - 75)
Sex Histology	Male Female Adenocarcinoma Mucinous adenocarcinoma	68 (58.6) 48 (41.4) 108 (93.1) 8 (6.9)
Distance from anal verge (cm)	≤ 5 cm > 5 cm	38 (32.8) 78 (67.2)
Initial CEA level (ng/ml)	Median (range) ≤ 5 > 5	2.9 (1-43) 81 (69.8) 35 (30.2)
AJCC stage, 7th ed.	IIIA IIIB IIIC	12 (10.3) 64 (55.2) 40 (34.5)
Pathologic tumor stage	pT2 pT3	15 (12.9) 101 (87.1)
Pathologic node stage	pN1 pN2	76 (65.5) 40 (34.5)
Type of surgery	LAR APR	95 (81.9) 21 (18.1)
Resection margin	Positive Negative	3 (2.6) 113 (97.4)
Differentiation	Well-differentiated (G1) Moderately-differentiated (G2) Poorly-differentiated (G3) Not evaluated (GX)	5 (4.3) 99 (85.3) 3 (2.6) 9 (7.8)
Lymphovascular space invasion	No Yes	85 (73.3) 31 (26.7)
Perineural invasion	No Yes	106 (91.4) 10 (8.6)
Postoperative radiation	Median dose Range	45 Gy 45 – 51 Gy
Concurrent chemotherapy	5-Fluorouracil 5-FU/LV	103 (88.8) 13 (11.2)

Abbreviations. CEA, carcinoembryonic antigen; AJCC, American Joint Committee on Cancer; LAR, low anterior resection; APR, abdomino-perineal resection; FU/LV, 5-fluorouracil + leucovorin

### Patterns of recurrence and survival outcome

The median follow-up duration of all patients was 119.0 months (range, 6.6 to 161.3) and 125.4 months for survivors at last follow-up (range, 82.5 to 161.3). During follow-up, tumor recurrence of any type was detected in 50 patients (43.1%). LR developed as a first failure site in 13 patients (11.2%) and DM in 41 patients (35.3%). Four of these patients experienced simultaneous LR and DM. The lung was the most frequent site of DM, occurring in 21 patients (18.1%), followed by the liver in 17 patients (14.7%), including combined liver and lung metastasis in 4 patients.

There were 40 deaths, including 4 cases of intercurrent death caused by ischemic heart disease and other primary malignancies, among other causes. OS, DFS, DMFS, and LRFS rates of all patients were 75.0%, 61.2%, 68.6%, and 87.4% at 5 years, respectively.

#### Expression of survivin and p53

Expression levels and subcellular localization of survivin and p53 were determined by IHC. Representative results of the expression profiling are summarized in Table 2. We applied the ‘minimum *p*-value’ approach to obtain a cutoff value for each antibody that provided the best separation between the groups of patients related to DFS and OS. Based on this approach, an immunostaining score of 80 or more was regarded as positive for survivin, and a score of 20 or more was defined as positive for p53.

**Table 2:** Expression of immunohistochemical markers in rectal cancer with lymph node metastasis

Expression of markers	Subcellular localization			Total Positive Rate
	Nucleus only	Cytoplasm only	Nucleus + Cytoplasm	
Survivin	23 (19.8%)	26 (22.4%)	7 (6.0%)	56/116 (48.3%)
P53	58 (50.0%)	0	0	58/116 (50.0%)

Positive immunostaining for nuclear and cytoplasmic survivin was observed in 30 patients (25.9%) and 33 patients (28.4%), respectively, with individual immunostaining scores ranging from 0 to 180. Nuclear staining for p53 was observed in 58 patients (50.0%), with scores ranging from 0 to 95 (Table 2). Seven patients (6.0%) showed positive expression of both nuclear and cytoplasmic survivin, and three patients (2.6%) had co-expression of all three molecular markers.

We analyzed correlations among the aberrant expression of each protein and found no significant correlations between the three molecular markers (for nuclear and cytoplasmic survivin, Pearson chi-square test, *p* = 0.471 as a dichotomized variable, Spearman's correlation *p* = 0.208 as a continuous variable; for nuclear survivin and p53, Pearson chi-square test, *p* = 0.396, Spearman's correlation, *p* = 0.724; for cytoplasmic survivin and p53, Pearson chi-square test, *p* = 0.065, Spearmans' correlation, *p* = 0.465).

#### Probable pognostic factors of recurrence

**Figure 1:**
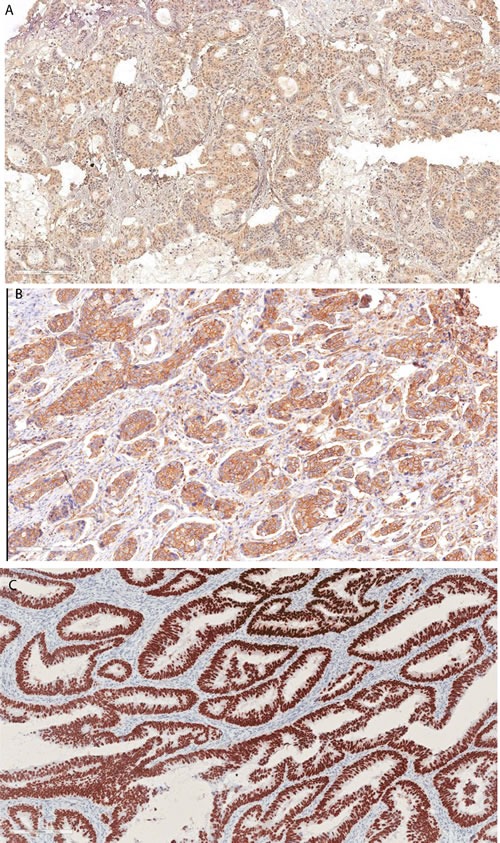
Representative immunohistochemical staining of survivin and p53 Positive survivin immunoreactivity in tumor cells (nuclear survivin, **A**; cytoplasmic survivin, **B**) and positive p53 expression in tumor cells (**C**).

Table 3 illustrates LRFS, DMFS, and DFS according to probable clinical, pathologic, and IHC prognostic factors. Patients with positive nuclear or cytoplasmic survivin and p53 had an increased probability of disease recurrence compared to those with negative staining (5-year DFS 33.3% *vs*. 70.9%, *p* = 0.001 for nuclear survivin; 45.5% *vs*. 67.4%, *p* = 0.003 for cytoplasmic survivin; 48.2% *vs*. 74.1, *p* = 0.03 for p53). In particular, these factors were significantly related with DMFS, except for nuclear survivin, which was also significantly correlated with LRFS (5-year LRFS 71.7% *vs*. 92.4%, *p* = 0.01). Survival curves according to survivin or p53 expression are displayed in Figure 1.

**Table 3:** Univariate analysis of probable prognostic factors in local recurrence-free survival (LRFS), distant metastasis-free survival (DMFS), and disease-free survival (DFS)

Variables	LRFS			DMFS				DFS	
	HR	95% CI	*P*	HR	95% CI	*P*	HR	95% CI	*P*
Age (years) ≤ 55 *vs.* > 55	0.85	0.29-2.53	0.77	1.31	0.71-2.42	0.40	1.20	0.69-2.09	0.52
Sex Feale *vs.* Male	1.73	0.58-5.14	0.33	0.81	0.43-1.53	0.51	1.06	0.60-1.85	0.85
Distance from anal verge ≤ 5 cm *vs.* > 5cm	0.50	0.17-1.50	0.22	0.44	0.24-0.82	0.009	0.52	0.30-0.91	0.02
Initial CEA level ≤ 5 ng/ml *vs.* > 5 ng/ml	1.38	0.45-4.21	0.58	1.62	0.86-3.04	0.13	1.69	0.96-2.97	0.07
Pathologic tumor stage T2 *vs.* T3	1.81	0.24-13.96	0.57	0.86	0.43-2.78	0.86	1.42	0.56-3.57	0.46
Pathologic node stage N1 *vs.* N2	5.25	1.61-17.10	0.006	2.07	1.12-3.83	0.02	2.48	1.42-4.33	0.001
Type of surgery LAR *vs.* APR	2.40	0.74-7.81	0.15	2. 95	1.52-5.72	0.001	2.87	1.56-5.27	0.001
Resection margin Negative *vs.* Positive	3.33	0.43-25.75	0.25	2.48	0.60-10.32	0.21	2.95	0.92-9.48	0.07
Lymphovascular invasion No *vs.* Yes	3.50	1.18-10.42	0.02	1.17	0.57-2.39	0.66	0.87	0.47-1.58	0.64
Perineural invasion No *vs.* Yes	1.78	0.39-8.03	0.45	1.27	0.45-3.56	0.65	1.30	0.52-3.29	0.57
Chemotherapy regimen 5-FU *vs.* FL	0.04	0.00-59.63	0.39	0.59	0.18-1.91	0.38	0.63	0.23-1.75	0.37
*P53* ≤20 *vs.* >20	1.36	0.46-4.04	0.59	2.37	1.24-4.52	0.009	1.88	1.07-3.31	0.03
Nuclear survivin ≤80 *vs.* >80	3.80	1.06-9.81	0.04	2.05	1.09-3.88	0.03	2.52	1.43-4.45	0.001
Cytoplasmic survivin ≤80 *vs.* >80	1.32	0.41-4.29	0.64	2.71	1.46-5.03	0.002	2.30	1.30-4.05	0.004

Abbreviations. DFS, disease-free survival rate; CEA, carcinoembryonic antigen; LAR, low anterior resection; APR, abdomino-perineal resection; AJCC, American Joint Committee on Cancer

Pathologic node stage, type of surgery, and distance from the anal verge were also significant prognostic factors in both DFS and DMFS. Pathologic node stage and lymphovascular invasion were significant prognostic factors for LRFS.

As illustrated in Table 4, multivariate analysis using Cox proportional hazard modeling showed that both nuclear and cytoplasmic survivin had a significant adverse effect on DMFS (nuclear survivin; HR 2.11, 95% CI 1.08-4.12, *p* = 0.03, cytoplasmic survivin; HR 2.65, 95% CI 1.35-5.20, *p* = 0.005) and DFS (nuclear survivin; HR 2.46, 95% CI 1.35-4.45, *p* = 0.003, cytoplasmic survivin; HR 2.16, 95% CI 1.17-4.00, *p* = 0.01). Nuclear survivin was also significantly related with LRFS (HR 3.23, 95% CI 1.06-9.81, *p* = 0.04). However, no survival difference was noticed according to p53 expression for DMFS (*p* = 0.08) or DFS (*p* = 0.17).

**Table 4:** Multivariate analysis of probable prognostic factors in disease free survival

Variables	LRFS			DMFS				DFS	
	HR	95% CI	*P*	HR	95% CI	*P*	HR	95% CI	*P*
Distance from anal verge ≤ 5 cm *vs.* > 5cm	-			1.28	0.53-3.11	0.58	0.96	0.42-2.21	0.93
Pathologic node stage N1 *vs.* N2	3.48	1.03-11.70	0.04	1.36	0.69-2.69	0.38	1.90	1.04-3.47	0.04
Type of surgery LAR *vs.* APR	-			2.18	0.87-5.49	0.10	2.73	1.14-6.56	0.02
Lymphovascular invasion No *vs.* Yes	2.78	0.91-8.49	0.07	-			-		
*P53* ≤20 *vs.* >20	-			1.86	0.92-3.77	0.08	1.54	0.83-2.89	0.17
Nuclear survivin ≤80 *vs.* >80	3.23	1.06-9.81	0.04	2.11	1.08-4.12	0.03	2.46	1.35-4.45	0.003
Cytoplasmic survivin ≤80 *vs.* >80	-			2.65	1.35-5.20	0.005	2.16	1.17-4.00	0.01

Abbreviations. HR, hazard ratio; 95% CI, 95% confidence interval; CEA, carcinoembryonic antigen; AJCC, American Joint Committee on Cancer; LAR, low anterior resection; APR, abdomino-perineal resection.

Multivariate analysis was performed with only significant prognostic factors in the univariate analysis (LRFS: Pathologic node stage, Lymphovascular invasion, Nuclear survivin; DMFS: Distance from anal verge, Pathologic node stage, Type of surgery, *P53*, Nuclear survivin, Cytoplasmic survivin; DFS: Distance from anal verge, Pathologic node stage, Type of surgery, *P53*, Nuclear survivin, Cytoplasmic survivin).

The other significant prognostic factors in DFS were pathologic node stage (HR 1.90, 95% CI 1.04-3.47, *p* = 0.04) and type of surgery (HR 2.73, 95% CI 1.14-6.56, *p* = 0.02).

#### Prognostic model of DFS

A prognostic model was established on the basis of the molecular marker survivin. Predictive grouping using IHC for cytoplasmic and nuclear survivin was performed according to the following criteria: group 1, no aberrant expression; group 2, one molecular marker showing positivity; and group 3, all markers showing positivity.

**Figure 2:**
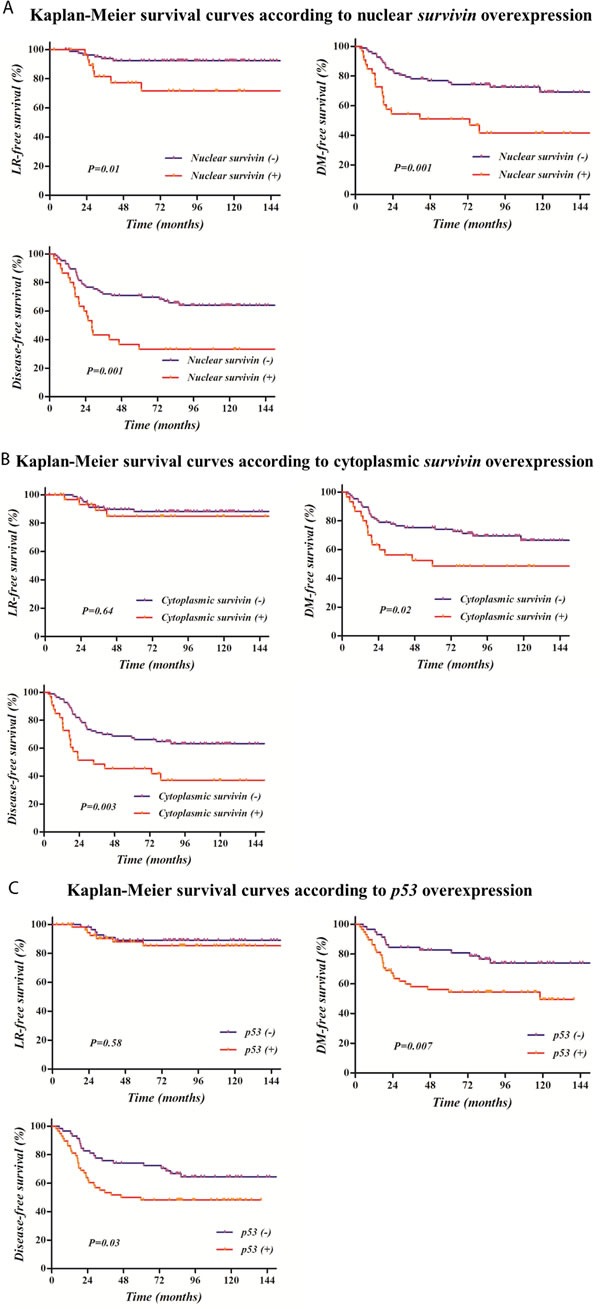
Kaplan-Meier survival curves according to survivin or p53 overexpression: Survival rates were significantly related with the overexpression of nuclear **A.** or cytoplasmic survivin **B.** and p53 **C.**, except for cytoplasmic survivin and p53 on local recurrence-free survival.

Survival curves according to the prognostic model based on *survivin* overexpression are shown in Figure 2, and the HR and 95% CI between the groups are displayed in Table 5. The three categorized groups had significantly different probabilities of disease recurrence (overall *p* < 0.001).

**Table 5:** Prognostic model of DFS according to the survivin overexpression

Prognostic group	survivin	n	HR	95% CI	*P*
Group 1	No (reference)	60			
Group 2	Nuclear or Cytoplasmic	49	3.41	1.45-5.17	0.004
Group 3	Both	7	5.25	1.63-13.12	0.02

The Cox proportional hazard model was used to determine the hazard ratio (HR) between groups respecting to DFS, and the results were adjusted with pathologic node stage and type of surgery which showed prognostic significance of DFS in multivariate analysis.

**Figure 3:**
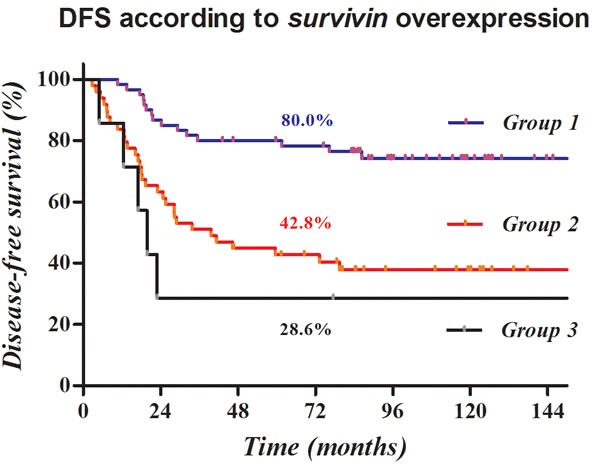
Kaplan-Meier survival curves according to a prognostic model based on survivin overexpression Disease-free survival was clearly stratified according to cytoplasmic or nuclear survivin overexpression, and most recurrences developed within two years.

## DISCUSSION

We evaluated the expression and prognostic significance of survivin and p53 in locally advanced rectal cancer treated with TME and postoperative CCRT. Positive immunostaining of nuclear and cytoplasmic survivin was observed in about one quarter of all patients, and p53 was found in half of all patients. Several expected clinical and pathological factors, including pathologic node stage and type of surgery, were confirmed to have significant roles as prognostic factors. Most of all, however, both cytoplasmic and nuclear survivin represented clear DFS differences, and the prognostic model established with cytoplasmic and nuclear survivin in locally advanced rectal cancer treated with TME and postoperative CCRT led to risk groups divided according to the recurrence risk in these patients.

The standard treatment for clinical T3 and/or N1-2 is preoperative CCRT followed by TME, but some portion of patients are diagnosed as T3 and/or N1-2 after curative resection, indicating postoperative CCRT [19]. Although clinical outcomes in these patients are persistently improving with adaptation and development of multimodality treatments such as anatomic surgery, conformal radiotherapy, and systemic chemotherapy, 30 to 40% of such cases recur within 3 years [1]. Although there are several recognized prognostic factors predicting recurrence [9–12], other reliable factors reflecting the biological behavior of the tumor could be used to apply more customized, patient-specific treatments with optimal risk-adapted surveillance after treatment. The importance of reliable prognostic factors is emphasized more in patients who are expected to have a favorable prognosis, and remains a controversial issue in determining the necessity and/or combination of adjuvant treatment, such as in patients with upper rectum confined tumors and/or a small amount of perirectal lymph node involved tumors.

The *TP53* gene, also known as the “guardian of the genome”, is a tumor suppressor gene that regulates cell cycle progression, DNA repair, cellular senescence, and apoptosis [20]. Mutations in TP53 are detected in more than 50% of human tumors [21]. When it is mutated, the p53 protein loses its tumor suppressing function. p53 represents the most widely studied gene in colorectal cancer based on various detection methods such as loss of heterozygosity, protein overexpression by IHC, and detection of mutations by either direct sequencing or single strand polymorphism analysis. IHC has provided enormous benefits in evaluating prognostic markers in neoplasms including colorectal cancer. It is considered a valuable diagnostic method with easy accessibility and well-established efficacy in examining the functional unit in a tumor cell. Despite a large number of publications and a systematic review, the impact of p53 abnormalities on prognosis in colorectal cancer remains uncertain [22]. In the present study, p53 protein overexpression appears to be a prognostic factor in rectal cancer, especially with respect to worse DFS, although statistical significance was not reached in multivariate analysis.

Survivin, also known as baculoviral inhibitor of apoptosis repeat-containing 5, is an inhibitor of apoptosis [18]. It has been reported that survivin is detected in several proliferating adult normal tissues, although it is more frequently reported in tumor and fetal tissues. It inhibits apoptosis or programmed cell death-related proteins such as caspase-3 and caspase-7, and also regulates cell division by localizing in the mitotic spindle and interacting with tubulin. There are many reports showing a clear relationship between the expression of survivin and dismal clinical outcomes in several sites of cancer [15–17, 23, 24].

Although several discrepancies have been noted, most studies have demonstrated that survivin is one of the most important prognostic factors in rectal cancer and have further suggested that it could be a critical factor of radio-resistance in cancer cells [17, 23, 24]. Rodel et al. proposed that survivin could serve as a predictive factor of radio-responsiveness based on a LR rate of 26% in the high survivin expression group compared with 6% in the low survivin expression group [17, 24]. In addition, Kim et al. reported that survivin may be related with radio-responsiveness in preoperative CCRT in rectal cancer, based on an association of survivin overexpression in pretreatment tumor biopsies with less tumor downstaging (25.7% *vs* 72.2% with low survivin expression, *p* = 0.001) [23]. In the postoperative CCRT setting, however, the significance of survivin expression remains uncertain. Furthermore, although it is known that survivin is localized in the nucleus and the cytoplasm [25], its roles based on subcellular localization have not yet been clarified, thereby its prognostic impact remains controversial.

In the present study, DFS was significantly related with the expression of survivin in locally advanced rectal cancer treated with TME followed by postoperative CCRT. In particular, overexpression of nuclear survivin showed an inverse relationship with LRFS. These results support the finding of Rodel and Kim et al. that the overexpression of nuclear survivin is related with radio-resistance of rectal cancer.

Overexpression of either nuclear or cytoplasmic survivin was clearly related to a higher risk of recurrence, especially DM, in the present study. Further, the risk of recurrence after TME followed by postoperative CCRT was clearly stratified by overexpression of survivin in both locations. In the presence of cytoplasmic or nuclear survivin overexpression, DFS dropped from 80.0% to 42.8% to 28.6% after 5 years. Most recurrences developed within two years, with DFS of 86.7%, 63.3%, and 28.6%, respectively.

More aggressive treatment to reduce recurrence and/or active surveillance for two years after treatment to detect early recurrence and facilitate salvage treatment will improve outcomes in high risk patients with survivin overexpression. Additionally, survivin might be a potentially valuable target for systemic treatment [26]. The pharmacologic and immunologic targeting of survivin overexpression in tumors may strongly enhance treatment outcomes. Based on these ideas, several prospective trials evaluating the efficacy of vaccines or agents targeting survivin are ongoing.

The present study had a number of limitations that warrant consideration. First, the single institutional retrospec­tive design carries with it inevitable concerns for selection bias. Second, the subjects of the present study were confined to stage III rectal cancer patients who received TME. Therefore, there should be careful interpretation of the outcomes in patients with other stages of cancer. Third, LRFS, DMFS, and DFS, which could be affected by the completeness of follow-up, were used as end points in the present study. Finally, the significance of survivin as a prognostic factor and prognostic models based on cytoplasmic and nuclear survivin were not validated with large, independent datasets.

Nevertheless, the present study provided highly useful information. The importance of either nuclear or cytoplasmic survivin as a prognostic factor for recurrence was verified in a relatively large number of cases of locally advanced rectal cancer patients treated with TME followed by postoperative CCRT. These findings aid in determination of the optimal adjuvant treatment plan, or surveillance in rectal cancer patients with confirmed pathologic T2-3 and N1-2 after TME without neoadjuvant treatment. Further large scale studies are needed to evaluate and validate the role of survivin as a prognostic factor in locally advanced rectal cancer.

## MATERIALS AND METHODS

### Selection of cases

Patients with histologically confirmed locally advanced rectal adenocarcinoma at Samsung Medical Center (Seoul, Korea) between June 2001 and September 2004 were enrolled in the present study. Eligibility criteria were (1) treatment with curative surgical resection, (2) pathologic T stage of 2 to 3 and/or regional lymph node metastasis, and (3) adjuvant CCRT with 5-fluorouracil (FU)-based chemotherapeutic agents. Patients who underwent preoperative chemotherapy and/or radiation therapy (RT), had pT4 or N0 disease, or received adjuvant chemotherapy with other than a 5-FU based regimen, such as capecitabine, were excluded from this study.

Staging work-up included comprehensive history, digital rectal exam, sigmoido- or colonoscopy with or without endorectal ultrasonography, simple chest X-ray, and contrast enhanced computed tomography (CT) and/or magnetic resonance imaging (MRI) of the abdomen and pelvis.

Treatment procedures consisted of curative TME and adjuvant CCRT. Surgical procedures comprised low anterior resection (LAR) or abdomino-perineal resection (APR). Adjuvant treatment, which was scheduled within 4 to 6 weeks after surgery, consisted of RT with concomitant application of 5-FU based chemotherapy.

Adjuvant RT was delivered to the whole pelvis with a dose of 45 gray (Gy) in 25 fractions. The radiation field encompassed a volume that included the anastomotic site, mesorectum, and regional area, including perirectal, presacral, and internal iliac lymphatics. An additional boost to the primary tumor bed was also given in some patients with evidence of adverse pathologic features such as a close/positive surgical resection margin.

Postoperative chemotherapy generally consisted of intravenous bolus 5-FU (500 mg/m^2^/day) with or without a combination with leucovorin (20 mg/m^2^/day) for five consecutive days every four weeks through a total of six cycles. Pelvic RT was generally administered during the third chemotherapy cycle. During CCRT, two cycles of bolus 5-FU (500 mg/m2/day) were administered for three days matched with the RT start and end date. In patients with a positive resection margin, RT was started with the first cycle of chemotherapy.

Follow-up included physical examination with digital rectal exam, blood tests for carcinoembryonic antigen (CEA), and CT scans of the abdomen and pelvis and/or colonoscopy every 3 months for 2 years after surgery, and then every 6 months thereafter.

### IHC and interpretation

Core tissue samples (2 mm in diameter) were obtained from surgical specimens, which were formalin fixed, paraffin embedded, and arranged in a new recipient paraffin block for tissue array. Four-μm sections were deparaffinized and dehydrated. IHC staining with anti-survivin antibody (dilution 1:400, Cell Signaling, Boston, MA, USA) and anti-p53 antibody (dilution 1:50; VECTOR, Burlingame, CA, USA) was performed. Appropriate positive and negative control samples were included for each run.

Cancer cells demonstrating nuclear or cytoplasmic staining were interpreted as positive immunostaining for survivin. For p53, only nuclear staining of cancer cells was considered an appropriate IHC staining pattern. Immunostaining of survivin and p53 was scored in a semiquantitative manner. Survivin expression was analyzed by an individual score considering the percentage of positive cells and staining intensity. Intensity was scored as follows: 0 (no stain), 1+ (weak), or 2+ (intense). Scores for survivin were estimated by the method of multiplication with staining intensity and percentage of positive cells, ranging from 0 to 200. The score for p53 was calculated according to the proportion of positive cells, ranging from 0 to 100. For statistical analysis, the specimens with an immunostaining score greater than 80 for survivin were grouped as positive immunoreactivity. For p53, no staining or staining in less than 20% of the tumor cells was defined as the loss of p53.

### Statistical analysis

First site recurrence was classified as local recurrence (LR, defined as recurrence inside the pelvic cavity at the anastomosis site, presacral area, or pelvic lymph node) and/or distant metastasis (DM, defined as recurrent tumor outside the pelvis). Overall survival (OS), DFS, DM-free survival (DMFS) and LR-free survival (LRFS) duration were calculated from the date of surgery to the date of event development or the latest documented follow-up. Survival rates were calculated using the Kaplan-Meier method. The Cox proportional hazard model was used to determine the independent prognostic factors significantly impacting survival, and only significant prognostic factors in the univariate analysis were used in the multivariate analysis. A *p*-value < 0.05 was regarded as statistically significant in two-tailed tests. Variable risk was expressed as a hazard ratio (HR) with a corresponding 95% confidence interval (CI). Bonferroni's method was used for multiple testing. Statistical analysis was performed using SPSS software (IBM SPSS Statistic version 22.0, Chicago, IL, USA).

## Abbreviations

TTME: total mesorectal excision
CCRT: postoperative concurrent chemo-radiation therapy
DFS: disease-free survival
IHC: immunohistochemistry
5-FU: 5-fluorouracil
CT: computed tomography
MRI: magnetic resonance imaging
LAR: low anterior resection
APR: abdomino-perineal resection
LR: local recurrence
DM: distant metastasis
CEA: carcinoembryonic antigen
OS: overall survival
DMFS: distant metastasis-free survival
LRFS: local recurrence-free survival
HR: hazard ratio
CI: confidence interval
